# Integrating Cortical Source Reconstruction and Adversarial Learning for EEG Classification

**DOI:** 10.3390/s25164989

**Published:** 2025-08-12

**Authors:** Yue Guo, Yan Pei, Rong Yao, Yueming Yan, Meirong Song, Haifang Li

**Affiliations:** 1 College of Computer Science and Technology (College of Data Science), Taiyuan University of Technology, Jinzhong 030600, China; guoyue@sxtbu.edu.cn (Y.G.); peiyan0433@link.tyut.edu.cn (Y.P.); yaorong@tyut.edu.cn (R.Y.); yanyueming0084@link.tyut.edu.cn (Y.Y.); 2024319018@link.tyut.edu.cn (M.S.); 2 College of Computer Information Engineering, Shanxi Technology and Business University, Taiyuan 030006, China

**Keywords:** EEG, depression classification, CFE strategy, GCN, domain adaptation

## Abstract

Existing methods for diagnosing depression rely heavily on subjective evaluations, whereas electroencephalography (EEG) emerges as a promising approach for objective diagnosis due to its non-invasiveness, low cost, and high temporal resolution. However, current EEG analysis methods are constrained by volume conduction effect and class imbalance, both of which adversely affect classification performance. To address these issues, this paper proposes a multi-stage deep learning model for EEG-based depression classification, integrating a cortical feature extraction strategy (CFE), a feature attention module (FA), a graph convolutional network (GCN), and a focal adversarial domain adaptation module (FADA). Specifically, the CFE strategy reconstructs brain cortical signals using the standardized low-resolution brain electromagnetic tomography (sLORETA) algorithm and extracts both linear and nonlinear features that capture cortical activity variations. The FA module enhances feature representation through a multi-head self-attention mechanism, effectively capturing spatiotemporal relationships across distinct brain regions. Subsequently, the GCN further extracts spatiotemporal EEG features by modeling functional connectivity between brain regions. The FADA module employs Focal Loss and Gradient Reversal Layer (GRL) mechanisms to suppress domain-specific information, alleviate class imbalance, and enhance intra-class sample aggregation. Experimental validation on the publicly available PRED+CT dataset demonstrates that the proposed model achieves a classification accuracy of 85.33%, outperforming current state-of-the-art methods by 2.16%. These results suggest that the proposed model holds strong potential for improving the accuracy and reliability of EEG-based depression classification.

## 1. Introduction

Depression has become one of the most prevalent and widespread psychiatric disorders worldwide. Patients typically exhibit symptoms such as a depressed mood, slowed thinking, and significant cognitive impairment, and severe cases may involve self-harm behaviors or life-threatening conditions [1,2]. Neuroimaging studies have confirmed that depression is associated with functional abnormalities in the prefrontal-limbic neural circuitry, particularly involving disrupted functional connectivity in regions such as the anterior cingulate cortex and dorsolateral prefrontal cortex. These neurophysiological alterations result in persistent deficits in emotional regulation and cognitive processing, and are also closely linked to the high recurrence rate of depression. According to the World Health Organization, depression currently affects more than 350 million people globally and is projected to become the leading cause of disease burden by 2030 [3,4,5]. With its rising prevalence, early diagnosis and intervention for depression are becoming increasingly critical.

Currently, depression diagnosis primarily relies on clinicians’ subjective assessments, often based on standardized symptom rating scales [6]. However, this approach has inherent limitations and may result in misdiagnosis or missed diagnoses [7,8]. Consequently, researchers have been actively exploring objective diagnostic methods. In recent years, advancements in neuroimaging technologies—such as EEG, functional magnetic resonance imaging (fMRI), and positron emission tomography (PET)—have provided powerful tools for studying and diagnosing depression [9,10]. These techniques allow for the monitoring and analysis of brain activity from different perspectives, offering more objective diagnostic criteria [11]. Nevertheless, practical challenges such as high costs and complicated equipment requirements have restricted their widespread accessibility and application.

In contrast, EEG, as a non-invasive, low-cost modality with exceptionally high temporal resolution, has emerged as a promising direction in research aimed at diagnosing brain disorders, particularly depression [12,13]. EEG-based depression classification methods generally fall into two categories: the first relies on handcrafted feature extraction combined with traditional machine learning algorithms [14,15,16,17]. Although this approach has achieved progress in certain scenarios, its effectiveness remains limited due to the inherently low spatial resolution and inter-individual variability of EEG signals. In recent years, deep learning techniques have demonstrated increasing advantages in EEG processing, significantly enhancing accuracy and robustness in depression classification through automated feature learning and high-dimensional data representation [18,19,20,21]. Deep learning methods overcome the limitations of traditional approaches through end-to-end training, promoting EEG application in depression classification tasks.

Despite valuable contributions from existing EEG-based depression classification methods, two key challenges remain inadequately addressed, which are outlined below.

First, the low spatial resolution of EEG signals restricts the capture of spatiotemporal features. Many studies extract features directly from scalp EEG signals. For instance, Acharya et al. [22] proposed a method for constructing a Depression Diagnosis Index based on multiple nonlinear features, which, combined with a Support Vector Machine (SVM), achieved high-accuracy automatic classification of depression. Zhang et al. [23] developed a brain functional network based on resting-state EEG and employed a Random Forest classifier, reaching a maximum classification accuracy of 93.31%. Yang et al. [24] introduced a fusion approach that combined Lempel-Ziv complexity features under both eyes-open and eyes-closed paradigms, and applied multiple classifiers for cross-subject depression recognition, achieving an accuracy of 94.03% using an SVM. Liu et al. [25] proposed a depression classification method combining spatiotemporal features, utilizing a GCN and an adjacency matrix based on channel correlations for resting-state EEG processing. Ying et al. [26] introduced the EEG-based Depression Transformer, extracting temporal, spatial, and frequency domain features to distinguish depressed patients from healthy controls. Seal et al. [27] developed a deep learning model based on convolutional neural networks (CNN), which achieved an accuracy of 99.37% on their private dataset. Other studies, such as those by Liu et al. [28] and Lu et al. [29], have also employed deep learning architectures to automatically extract spatiotemporal features, achieving impressive classification performance. However, the volume conduction effect of EEG often leads to pseudo-connectivity and inaccurate regional information [30,31,32], making it difficult for existing feature extraction methods to fully capture the spatiotemporal characteristics of EEG signals.

Second, class imbalance negatively impacts classification effectiveness. Due to the significant disparity in sample sizes between depressed patients and healthy controls, many studies have focused on improving model generalizability. For example, Ye et al. [33] integrated deep similarity learning and adversarial learning, proposing a cross-subject emotional recognition method. Song et al. [34] combined CNN with LSTM, employing a domain discriminator to reduce differences between training and testing datasets. Jia et al. [35] proposed the MSTGCN model with domain generalization capability to extract subject-invariant sleep features. Additionally, Mohammed et al. [36] applied domain adaptation techniques to mitigate inter-subject feature distribution discrepancies, enhancing depression classification performance. He et al. [37] designed three alignment mechanisms—domain alignment, semantic alignment, and structural alignment—within a deep neural network framework to minimize domain gaps. Jin et al. [38] proposed a method that combined unsupervised semantic segmentation with multi-level feature space adversarial transfer learning, significantly improving localization accuracy and segmentation quality in real-world scenarios. Ayodele et al. [39] employed a domain generalization strategy to integrate multi-source EEG data and utilized a recurrent convolutional network for epilepsy detection, achieving 72.5% sensitivity and a low false positive rate on an independent dataset. Ganin et al. [40] proposed a deep domain adaptation method incorporating a GRL, which enabled joint training on labeled source-domain data and unlabeled target-domain data, thereby promoting the learning of discriminative and domain-invariant features. Li et al. [41] employed a convolutional neural network integrated with transfer learning to recognize mild depression, leveraging spectral, spatial, and temporal information from EEG signals. However, existing studies generally overlook the class imbalance problem, potentially a critical factor contributing to suboptimal classification performance.

Therefore, this paper proposes a multi-stage deep learning model for EEG-based depression classification, which integrates a CFE strategy, FA module, GCN, and FADA module to improve classification accuracy and generalizability. Specifically, the CFE strategy reconstructs brain cortical source signals using the sLORETA algorithm and extracts multi-dimensional features to characterize cortical activity. The FA module leverages a multi-head self-attention mechanism to enhance the representation of spatiotemporal dependencies among brain regions. The GCN models functional connectivity between brain regions to capture higher-order structural information, while the FADA module alleviates class imbalance and domain shift issues through the use of Focal Loss and GRL mechanisms. Experimental validation on the publicly available PRED+CT dataset demonstrates that the proposed model achieves an accuracy of 85.33%, representing a 2.16% improvement over current mainstream methods, thereby highlighting its effectiveness and potential in cross-subject EEG analysis for depression classification.

## 2. Methods

This section first provides an overview of the proposed model framework, followed by detailed descriptions of the CFE strategy, FA module, and GCN. Finally, this paper discusses the FADA module to enhance the model’s robustness and generalization capability.

### 2.1. Overall Framework of Our Model

**Figure 1:**
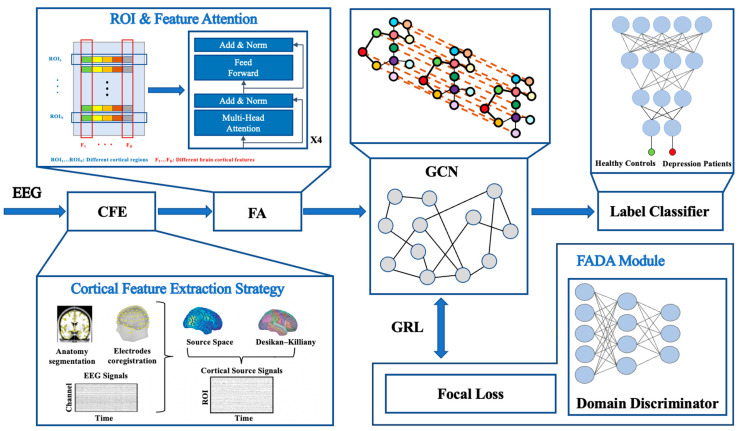
Framework of the proposed depression classification model. EEG signals are first mapped to cortical source space, extracting high-resolution brain cortical features (CFE), enhanced through a multi-head attention mechanism (FA). Then, a GCN integrates cortical connectivity to extract deep features for classification. Meanwhile, the FADA module with Focal Loss is introduced to enhance cross-subject generalization and discrimination of challenging samples.

Figure 1 illustrates the overall framework of the proposed depression classification model. First, raw multi-channel EEG signals are processed through the CFE strategy, mapping EEG signals to high-resolution cortical source signals using the sLORETA algorithm. Subsequently, multi-dimensional features including frequency-domain, time-domain, spatial, and nonlinear characteristics are extracted, forming feature matrices that reflect cortical activation patterns. Next, brain cortical features enter the FA module, which is based on a Transformer architecture, employing a multi-head attention mechanism to enhance key regional and temporal features. A GCN, integrated with the cortical connectivity graph, further models structured high-dimensional features to extract discriminative representations. Finally, classification is performed using a classifier. To enhance the model’s generalization ability, the FADA module employs a GRL to mask domain-specific information and incorporates Focal Loss to balance the samples.

### 2.2. Cortical Feature Extraction Strategy

The CFE strategy aims to extract biologically meaningful brain cortical features from EEG signals, reflecting cortical activities relevant for depression classification. A two-step procedure is adopted: firstly, cortical source signals are reconstructed using sLORETA [42] to derive current-source distributions; secondly, multi-dimensional features are extracted from cortical signals across different frequency bands, encompassing temporal, spectral, spatial, and nonlinear characteristics.

#### 2.2.1. Cortical Source Reconstruction

Cortical source signals are reconstructed via sLORETA using the Brainstorm toolbox (v3.4) in MATLAB R2021a [43]. The reconstruction process begins by building a four-layer boundary element head model, comprising the scalp, outer skull, inner skull, and cortex [44]. This model is based on the ICBM152 MNI standard template, defining a cortical source space with 15,002 vertices (~5 mm spacing). Each vertex is represented by a current dipole oriented perpendicular to the cortical surface.

Source reconstruction solves the EEG inverse problem, inferring cortical source distributions $J\in R^{S\times T}$ (source points S, time points T) from scalp potentials $\Phi \in R^{L\times T}$ (electrodes L). The process is described by the lead field matrix $K\in R^{L\times S}$:

(1) Φ=KJ+ϵ

where ϵ denotes measurement noise. Due to the severe ill-posedness of the inverse problem, sLORETA introduces Tikhonov regularization:

(2) $$\hat{J}=arg\underset{J}{min}\left\{∥\Phi -KJ{∥}_{F}^{2}+\alpha ∥J{∥}_{F}^{2}\right\}$$

where $\|\cdot {\|}_{F}$ denotes the Frobenius norm and the closed-form solution of the formula is

(3) $$\hat{J}=K^{⊤}{\left(KK^{⊤}+\alpha I\right)}^{-1}\Phi$$

where I denotes the identity matrix. The regularization parameter α is estimated using generalized cross-validation to balance the data-fitting term and the norm constraint of the solution. To eliminate the dependence of source localization on current amplitude, sLORETA standardizes the current density:

(4) $$J_{\mathrm{sLORETA},i}=\frac{{\hat{J}}_{i}}{\sqrt{{\hat{J}}_{i}^{⊤}{\left(T_{i}\Sigma T_{i}^{⊤}\right)}^{-1}{\hat{J}}_{i}}}$$

where ${\hat{J}}_{i}\in R^{1\times 1}$ is the estimated dipole moment at source i, Σ denotes the noise covariance (usually $\sigma^{2}I$), and $T_{i}$ is the transfer submatrix for source i.

The sLORETA implementation is performed using the Brainstorm toolbox (v3.4) within MATLAB R2021a. The electrode-level EEG data Φ, recorded via the standard 10–20 electrode system, were spatially co-registered with the head model to construct the forward model. A four-layer boundary element method (BEM) was utilized for the head model, comprising scalp, outer skull, inner skull, and cortical surfaces, with the cortical surface serving as the defined source space J. The cortical source space, constructed based on the ICBM152 template, contains 15,002 vertices, each assigned a dipole source oriented perpendicular to the cortical surface. The lead-field matrix K was generated using the aforementioned BEM model, with conductivity values assigned as 1 S/m for cerebrospinal fluid, 0.0125 S/m for the skull, and 1 S/m for the scalp. The regularization parameter α was set to 0.05 to balance data-fitting and source-norm constraints. After source estimation, the vertex-level current densities $\hat{J}$ were mapped onto 68 regions of interest (ROIs) defined by the Desikan–Killiany atlas [45], and the averaged current density across all vertices within each region was used as the final feature representation for that region. Details of the ROIs are provided in Table 1.

**Figure 2:**
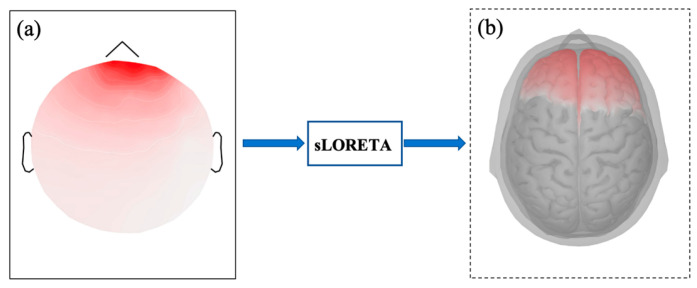
The brain topographic maps for source localization. (**a**) Scalp-level signal activity (**b**) Source-level signal activity.

Figure 2a illustrates the distribution of original EEG signals in the scalp space, while Figure 2b depicts the corresponding cortical source signals after source reconstruction.

#### 2.2.2. Feature Extraction

Based on evidence from EEG studies on depression, multi-dimensional features across frequency bands of ROI signals (theta 4–8 Hz, alpha 8–13 Hz, beta 13–30 Hz, gamma 31–80 Hz) are extracted, which contain linear features (spatial, temporal, and spectral domains) and nonlinear features (dynamic complexity metric), aiming to comprehensively characterize the EEG signal and provide effective input information for depression classification. The complete feature list is shown in Table 2.

Spatial features are extracted by constructing a brain network, where each ROI acts as a node and the connection strength between nodes is quantified using the Phase Locking Value (PLV) [46]. The PLV measures the degree of phase synchronization between a pair of signals in the frequency band f, which is computed as

(5) $${\mathrm{PLV}}_{pq}^{f}=\left|\frac{1}{T}\sum_{t=1}^{T}exp\left[i\left(\phi_{p}^{f}\left(t\right)-\phi_{q}^{f}\left(t\right)\right)\right]\right|$$

where $\phi_{p}^{f}\left(t\right)$ denotes the instantaneous phase of the signal in the frequency band f and time point t (obtained by Hilbert transform), and T is the total number of time points. The PLV ranges from 0 to 1, quantifying the degree of phase synchronization—where values closer to 1 indicate stronger phase synchronization.

Based on the PLV adjacency matrix, the Clustering Coefficient (Cp) and Local Efficiency (Eloc) are further calculated. The Cp measures the degree to which the neighbors of a node are interconnected, and is defined as

(6) $$Cp_{i}=\frac{2E_{i}}{k_{i}\left(k_{i}-1\right)}$$

where $E_{i}$ denotes the actual number of edges among the neighbors of node i, and $k_{i}$ is the degree (number of neighboring nodes) of node i. Eloc quantifies the efficiency of information transfer within the local subgraph composed of a node’s immediate neighbors, and is defined as

(7) $$Eloc_{i}=\frac{\sum_{j,h\in V,j\neq h}a_{ij}a_{ih}{\left[d_{jh}\left(N_{i}\right)\right]}^{-1}}{k_{i}\left(k_{i}-1\right)}$$

where $a_{ij}$ and $a_{ih}$ are the elements of the adjacency matrix (1 if the connection exists, 0 otherwise), and $d_{jh}\left(N_{i}\right)$ is the shortest path length through the neighbors of node i.

Finally, all brain cortical features extracted from different frequency bands and dimensions are organized into a feature matrix $X\in R^{N\times D}$, where N is the number of ROIs, and D is the sum of the feature dimensions extracted from each ROI. In this paper, D includes spectral features [47], temporal features, spatial features [48], and nonlinear features [49].

### 2.3. Feature Attention Module

To further enhance the feature representation of EEG data, we introduce the FA module based on the Transformer architecture. This module enhances the input features through a multi-head self-attention mechanism, aiming to effectively capture the spatiotemporal relationships between different brain regions and thus improve the accuracy of depression classification. The FA module employs a 4-layer multi-head self-attention mechanism, which is able to enrich feature representations while preserving the original feature dimensionality.

#### Module Architecture

The core of the FA module is a Transformer architecture based on the multi-head self-attention mechanism. In this architecture, each layer contains multiple parallel self-attentive heads, which independently capture relationships among input features within different subspaces. Specifically, the FA module takes as input the feature matrix $X\in R^{N\times D}$ of ROI signals, processes the features using the multiple self-attention mechanism, and outputs an enhanced feature matrix $\hat{X}\in R^{N\times D}$.

First, the input feature matrix X is mapped to the query, key, and value matrices by different linear transformations, which are expressed as

(8) $$Q=XW_{Q},\;K=XW_{K},\;V=XW_{V}$$

where $W_{Q},W_{K},W_{V}$ are learnable weight matrices, and Q,K,V denote the query, key, and value matrices, respectively. Next, the attention scores are computed and the value matrix is weighted and summed to generate the output for each header. The attention mechanism is computed as

(9) $$\mathrm{Attention}\left(Q,K,V\right)=\mathrm{softmax}\left(\frac{QK^{T}}{\sqrt{d_{k}}}\right)V$$

where $\sqrt{d_{k}}$ is the scaling factor and $d_{k}$ is the dimension of the key vectors. The outputs from all attention heads are concatenated and passed through a linear transformation to produce the final output matrix $\hat{X}$. The computation is formulated as

(10) $$\hat{X}=\mathrm{Concat}\left({\hat{X}}_{1},{\hat{X}}_{2},\ldots ,{\hat{X}}_{H}\right)W_{O}$$

where H denotes the number of attention heads, and $W_{O}$ is the linear transformation matrix of the output. By adding the input from the previous layer and applying a ReLU activation function, nonlinear transformations of the features are ensured, completing the processing of each layer.

The process is repeated for four layers, resulting in a deep feature enhancement network. The output of each layer is used as input to the next layer, thus capturing more complex feature relationships at multiple levels.

### 2.4. Graph Convolution Neural Network

GCN is a deep learning model specifically designed for processing graph-structured data, which can directly model the topological relationships among nodes in the data compared to traditional CNN. In this paper, GCN is used to process multi-dimensional features extracted from ROI signals, learning the functional connectivity relationships between brain regions, and to effectively capture spatiotemporal characteristics of neural activity through graph convolution operations.

#### 2.4.1. Feature Input and Adjacency Matrix Construction

In the GCN model, the input feature matrix $\hat{X}$ consists of the multi-dimensional features obtained from the CFE strategy described in the previous section. These features effectively reflect the spatiotemporal patterns in the ROI signals. To further capture the spatial structural relationships between brain regions, we construct an adjacency matrix A. Previous studies have shown that the correlation coefficient between different brain regions is an effective indicator for depression classification [50]. This adjacency matrix is constructed as follows:

(11) $$A_{mn}=\left\{\begin{array}{ll} 1 & \mathrm{if}\;Corr\left(x_{m},x_{n}\right)\geq \phi \\ 0 & \mathrm{otherwise} \end{array}\right.$$

where $Corr\left(x_{m},x_{n}\right)$ denotes the pearson correlation coefficient between the mth and nth ROI signals, and ϕ=0.3 is the predefined correlation threshold. If $Corr\left(x_{m},x_{n}\right)$ exceeds this threshold, then it is considered that there is a connection between the two ROIs, and the corresponding element $A_{mn}$ in the adjacency matrix is set to 1; otherwise, $A_{mn}$ is set to 0.

#### 2.4.2. Graph Convolution Operation

The graph convolution operation is the core mechanism of the GCN, integrating both the topological structure of the graph and the feature information of nodes, and is able to efficiently learn both local and global structural patterns among nodes. In this paper, the graph convolution operation is performed using the normalized adjacency matrix $\hat{A}$ and the feature matrix $\hat{X}$. The formula for graph convolution is as follows:

(12) $$H^{\left(l+1\right)}=\sigma \left(\hat{A}H^{\left(l\right)}W^{\left(l\right)}\right)$$

where $H^{\left(l\right)}$ denotes the input feature matrix of the graph convolution layer l, $H^{\left(l+1\right)}$ represents the output feature matrix of the graph convolution layer l+1, $\hat{A}$ is the normalized adjacency matrix, $W^{\left(l\right)}$ is the convolution kernel weights of the lth layer, and $\sigma \left(\cdot \right)$ is the activation function, and ReLU is used in this paper.

To avoid the adverse effects of large variations in node degrees in graph convolution on model training, the adjacency matrix A is normalized:

(13) $$\hat{A}=DM^{-1/2}\left(A+I\right)DM^{-1/2}$$

where A is the original adjacency matrix, I is the identity matrix, and DM is the degree matrix, $DM_{ii}=\sum_{j}A_{ij}$, which represents the degree of node i. The normalized adjacency matrix $\hat{A}$ ensures that the contribution of each node in the convolution operation is more balanced, which helps to improve the learning ability of GCN on graph-structured data.

### 2.5. Focal Adversarial Domain Adaptation Module

This paper proposes a module called FADA, which integrates Focal Loss, a domain-adversarial training mechanism based on GRL, and a class-center constraint (Center Loss) to construct a robust classification strategy for cross-subject EEG analysis. The FADA is designed to simultaneously address the class imbalance problem, achieve inter-domain feature alignment, and enhance the intra-class compactness in the feature space, thus adapting to the inherent distributional heterogeneity of EEG signals across different individuals.

#### 2.5.1. Focal Loss for Addressing Class Imbalance

In depression-related EEG data, significant class imbalance often arises due to limitations in clinical sample collection. During training, models tend to bias toward the majority class, which affects the accuracy of depression classification. The FADA introduces Focal Loss as the main classification loss function, which effectively improves the model’s ability to focus on minority class and boundary samples. It is defined as follows:

(14) $$L_{\mathrm{focal}}=-\sum_{i=1}^{M}\beta_{\tau_{i}}{\left(1-p_{\tau_{i}}\right)}^{\gamma }log\left(p_{\tau_{i}}\right)$$

where M denotes the total number of training samples, $x_{i}$ represents the ith sample, $\tau_{i}\in \{0,1\}$ denotes its ground-truth label (with 0 indicating a depressed subject and 1 indicating a healthy subject), and $p_{\tau_{i}}$ denotes the predicted probability for the true class. The parameter $\beta_{\tau_{i}}$ serves as a class-balancing factor, and γ is the focal parameter, which suppresses the model’s excessive attention to easily classified samples. Focal Loss significantly enhances the model’s ability to distinguish minority class instances, providing a stable foundation for subsequent domain alignment and intra-class compactness constraints.

#### 2.5.2. Adversarial Domain Adaptation Mechanism Based on GRL

EEG signals often exhibit substantial distributional shifts across individuals and environments, making it difficult for traditional supervised training to generalize to new domains. To address this issue, the FADA module integrates an adversarial domain adaptation mechanism based on GRL, aiming to encourage the model to learn domain-invariant discriminative features.

Let the feature extractor be $f_{\theta }$, the classifier be C, and the domain discriminator be D. The model is trained jointly through two optimization objectives: first, minimizing the classification loss to improve predictive performance; second, maximizing the domain discriminator loss so that the extracted features are difficult to distinguish from the source domain, thereby achieving cross-domain alignment.

The adversarial loss is defined as

(15) $$L_{\mathrm{adv}}=-\sum_{i=1}^{M}\sum_{k=1}^{K}I\left(d_{i}=k\right)logD\left(f_{\theta }\left(x_{i}\right)\right)$$

where $d_{i}$ denotes the domain label of sample $x_{i}$, K represents the total number of source domains, and $I\left(\cdot \right)$ is an indicator function used to determine whether a sample belongs to the kth source domain. The GRL connects the feature extractor and domain discriminator by reversing the gradient during backpropagation, resulting in the following update rule:

(16) $$\theta \leftarrow \theta -\eta \cdot \left(\frac{\partial L_{\mathrm{focal}}}{\partial \theta }-\lambda \cdot \frac{\partial L_{\mathrm{adv}}}{\partial \theta }\right)$$

where η is the learning rate, and λ is a hyperparameter that controls the strength of adversarial training. Through this mechanism, the FADA enables domain-adversarial optimization, enabling the feature extractor to learn more stable and transferable feature representations.

#### 2.5.3. Class-Center Constraint for Enhancing Intra-Class Compactness

After domain alignment, samples of the same class in different domains may still be loosely distributed, affecting the stability of classification boundaries. To address this issue, the FADA module further introduces class-centered loss, which constrains samples of the same class to cluster around the same class center to enhance intra-class consistency. The loss is defined as

(17) $$L_{\mathrm{center}}=\sum_{i=1}^{M}{∥f_{\theta }\left(x_{i}\right)-c_{\tau_{i}}∥}_{2}^{2}$$

where $f_{\theta }\left(x_{i}\right)$ denotes the feature representation of sample $x_{i}$, and $c_{\tau_{i}}$ is the center vector of class $\tau_{i}$. The norm term measures the distance between the feature and its corresponding class center. This loss term jointly optimizes the class center positions during training, guiding the model to learn discriminative and compact intra-class representations, which is particularly significant for pathological states with blurred boundaries, such as depression.

#### 2.5.4. Joint Optimization Objective

The FADA module comprehensively considers classification accuracy, domain alignment, and intra-class consistency to construct a unified joint loss function. The overall optimization objective is as follows:

(18) $$L_{\mathrm{FADA}}=L_{\mathrm{focal}}+\gamma \cdot L_{\mathrm{center}}-\lambda \cdot L_{\mathrm{adv}}$$

where γ and λ are the weight hyperparameters of class Center Loss and adversarial loss, respectively, which are used to balance the three objectives. This joint optimization strategy ensures that the model has the ability to cope with challenges such as class imbalance, cross-domain differences, and feature discreteness in EEG data for depression, thereby achieving stable and efficient classification performance.

## 3. Results and Discussion

This section first describes the dataset and preprocessing pipeline, followed by implementation details and comparative experimental results. Subsequently, ablation analyses of key modules are conducted to evaluate their contributions. Finally, statistical tests are employed to explore the electrophysiological significance of our model.

### 3.1. Participants

To evaluate the effectiveness of the proposed model, experiments are conducted on the publicly available PRED+CT dataset (https://openneuro.org/datasets/ds003478/versions/1.1.0 (accessed on 7 March 2025)). This dataset comprises resting-state EEG data from 122 college students [51], including 46 participants with depression or high Beck Depression Inventory (BDI) scores (≥13), and the remaining participants serve as healthy controls with low BDI scores (<7). Two healthy participants are excluded due to incomplete information. EEG recordings are approved by the Ethics Committee of Arizona State University, and informed consent is obtained from all participants. EEG signals are recorded using 64 Ag/AgCl electrodes placed according to the international 10–20 system and sampled at 500 Hz. To objectively assess model performance, no additional subject screening is performed. Relevant demographic information and the subscale scores derived from the BDI and the Trait Anxiety Inventory (TAI) are summarized in Table 3.

To increase the sample size, this paper adopts the same preprocessing strategy as Zhang et al. [52], whereby each participant’s EEG recording is divided into 150 epochs of 2 s each, resulting in 6900 epochs (46 subjects × 150) for depression patients (DP) and 11,100 epochs (74 subjects × 150) for healthy controls (HC).

### 3.2. Data Preprocessing

To mitigate the impact of data quality on model evaluation, EEG signals from the PRED+CT dataset undergo standardized preprocessing using the EEGLAB toolbox (https://sccn.ucsd.edu/eeglab/index.php (accessed on 5 March 2025)) in MATLAB. First, a 50 Hz notch filter is applied to remove power-line interference [53], followed by a band-pass filter retaining EEG frequencies of 4–80 Hz. The signals are then downsampled to 250 Hz to reduce data dimensionality while preserving essential information. Artifacts such as ocular and muscular noise are automatically identified and removed using the Faster algorithm [54]. Finally, EEG signals are standardized across channels via Z-score normalization:

(19) $$C^{*}=\frac{C-\mu }{\omega }$$

where C denotes the original EEG signal, $C^{*}$ is the normalized signal, and μ and ω represent the mean and standard deviation, respectively.

### 3.3. Implementation Details

All models are trained and evaluated on an NVIDIA RTX 4090 GPU (24 GB VRAM), an Intel i7-13700KF CPU, and 64 GB of RAM. Experiments are conducted using MATLAB R2021b, Python 3.9, and the PyTorch (v2.7.1) deep learning framework. Hyperparameter configurations are provided in Table 4.

Performance metrics employed for model evaluation included accuracy (ACC), precision (*PRE*), recall (*REC*), F1-score, and confusion matrices, computed as follows:

(20) $$ACC=\frac{TP+TN}{TP+TN+FP+FN}$$

(21) $$PRE=\frac{TP}{TP+FP}$$

(22) $$REC=\frac{TP}{TP+FN}$$

(23) $$F1-score=2\cdot \frac{PRE\cdot REC}{PRE+REC}$$

where *TP*, *TN*, *FP*, and *FN* denote true positives, true negatives, false positives, and false negatives, respectively. Confusion matrices further detailed these classifications.

### 3.4. Comparison with Other Methods

Our proposed model is systematically compared with existing methods on the PRED+CT dataset.

As shown in Table 5, our model demonstrates superior performance, achieving significant improvements in ACC (+2.16%) and PRE (+1.12%) compared to current state-of-the-art (SOTA) methods. The improvement in ACC is attributed to the enhanced feature accuracy provided by the CFE strategy and the effectiveness of Focal Loss in addressing difficult samples. The increase in PRE reflects the FADA module’s effectiveness in improving generalization and distinguishing between depressed and healthy subjects, which aligns with the findings reported in Zhang et al. [52].

**Figure 3:**
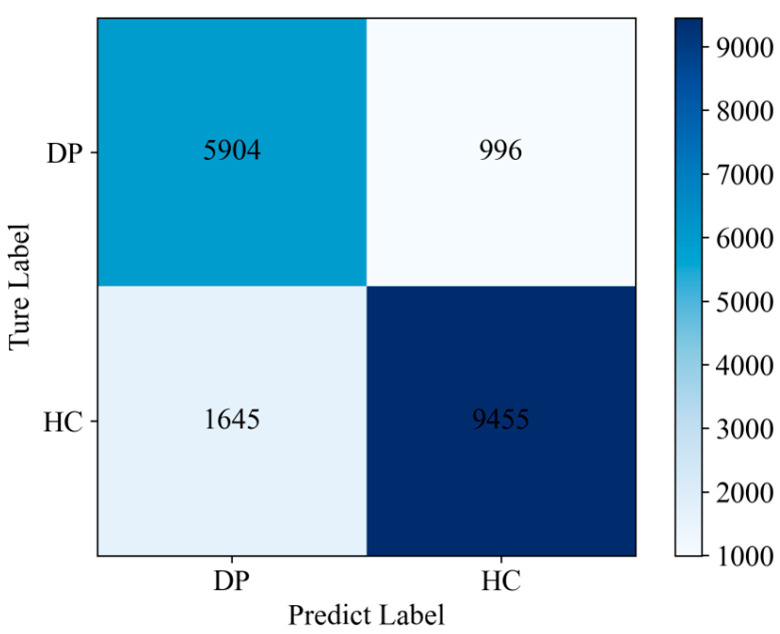
Confusion matrix of the proposed model.

Furthermore, the confusion matrix (Figure 3) indicates that the proposed model maintains a false positive rate (FPR) of 14.82% while achieving a true positive rate (TPR) of 85.57%.

In summary, the proposed model demonstrates strong classification performance on the PRED+CT dataset, validating its effectiveness and practical potential for EEG-based depression classification.

### 3.5. Ablation Experiment

This paper conducts extensive ablation experiments on the PRED+CT dataset (Table 6):

**CFE Strategy:** The CFE is primarily responsible for extracting cortical-level features. Comparing the baseline model (S1) and S2 shows that incorporating CFE significantly improves accuracy, with ACC increasing from 57.81% to 79.80%. This confirms that high-resolution cortical source signals substantially enhance classification performance, corroborating the findings of [59]. The improvement in REC (+7.59%) further highlights CFE’s contribution to feature granularity.

**FA Module:** As the sole attention mechanism, the FA effectively enhances cortical features. Comparing S1 and S3 shows that FA improves all classification metrics. Further comparison between S3 and S5 indicates a 6% improvement in ACC, suggesting complementary functionality between FA and CFE, with FA enhancing the features extracted by CFE.

**FADA Module:** Comparing S4 and S1 shows that all metrics improve, with REC notably increasing by 2.26%. The comparison between S8 and S5 reveals a marginal improvement in ACC (+1.83%). Relative to the baseline, S4 increases ACC by 1.19%, which is less significant than the improvements observed in S2 and S3. This suggests that enhancing feature granularity and representation is more effective than improving generalization alone in boosting classification performance.

**Figure 4:**
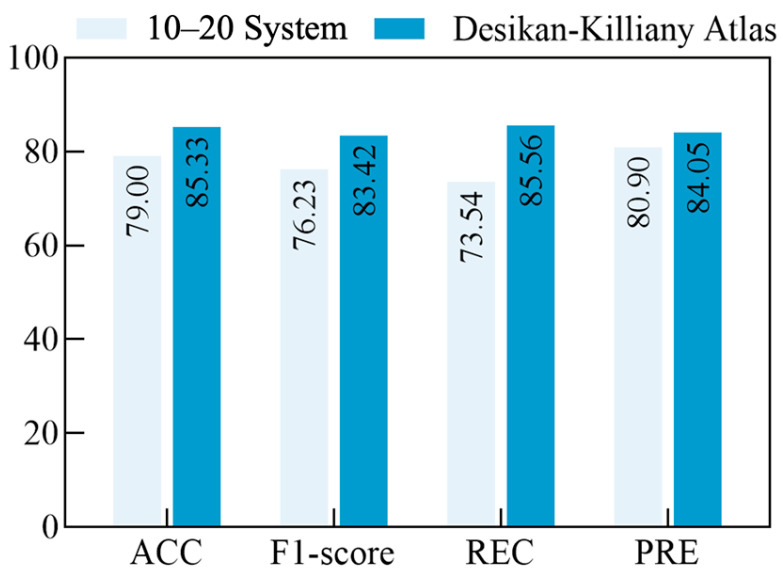
Ablation study on brain atlases.

**Brain Atlas Ablation:** A key innovation of this study lies in the source reconstruction of EEG signals to extract fine-grained cortical features, for which we conduct an ablation study comparing scalp-level features with brain cortical features. Figure 4 illustrates the comparative influence of the international 10–20 electrode system versus the Desikan–Killiany atlas. Brain cortical features significantly improve classification performance, increasing ACC by 6.33%, F1-score by 7.19%, REC by 12.02%, and PRE by 3.15%. Detailed statistical analysis is provided in Section 3.6.

**Figure 5:**
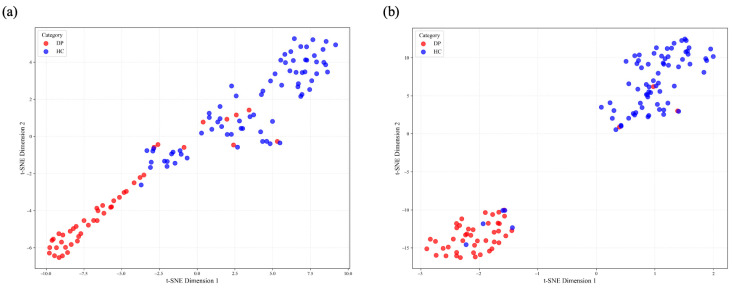
t-SNE visualization results. (**a**) t-SNE projection of the scalp features; (**b**) t-SNE projection of the source-space cortical features. Blue and red represent HC and DP samples, respectively.

In addition, Figure 5 illustrates the distributions of scalp features and source-space cortical features in a two-dimensional embedding space. Although the original scalp features (Figure 5a) show partial separation between the HC (blue) and DP (red) groups, a substantial overlap remains. In contrast, the source-space features (Figure 5b) present a clearer separation, with HC samples clustering in the upper-right region and DP samples in the lower-left region, leading to markedly reduced overlap.

### 3.6. Statistics and Analysis

**Figure 6:**
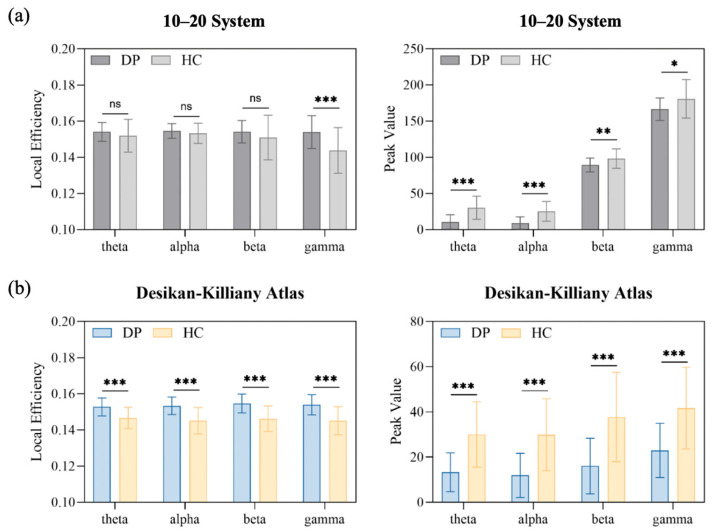
Group-level statistical results of scalp and brain cortical features. (**a**) Comparisons of scalp features based on the international 10–20 electrode system. The left panel shows local efficiency computed from PLV-based brain networks; the right panel shows the peak amplitude of EEG signals. (**b**) Comparisons of source-space brain cortical features based on the Desikan–Killiany atlas. The left panel presents local efficiency derived from PLV-based cortical networks; the right panel shows peak amplitude of source-reconstructed signals. Statistical significance is assessed using independent-sample *t*-tests. Significance levels are denoted as follows: ns = not significant (*p* ≥ 0.05); * *p* < 0.05; ** *p* < 0.001; *** *p* < 0.0001.

It has been shown that depression patients have frequency band specificity in neurophysiological features [60,61,62]. To further validate the effectiveness of the proposed CFE strategy for feature extraction, this section conducts independent-sample *t*-tests on representative features from the PRED+CT dataset under both scalp space (based on the international 10–20 electrode system) and source space (based on the Desikan–Killiany atlas). Figure 6a presents the group-level statistical results of scalp features, while Figure 6b shows the results for cortical features in the source space.

As shown in Figure 6a, the local efficiency of the DP group is generally higher than that of the HC group, while the peak value is generally lower. Among these, local efficiency shows a significant difference only in the gamma band (*p* < 0.0001), whereas the peak value exhibits statistically significant differences across all four frequency bands (theta: *p* < 0.0001, alpha: *p* < 0.0001, beta: *p* = 0.003, gamma: *p* = 0.01). Local efficiency reflects the efficiency of information transfer within local brain networks; an increase may indicate that information is being transmitted across a broader range of brain regions. This result aligns with the findings of Yi et al. [63], who report abnormal functional connectivity patterns in individuals with depression. Meanwhile, the reduced peak value in the DP group may reflect weakened cortical inhibition mechanisms, consistent with the findings of Wang et al. [64] on low-frequency amplitude abnormalities. A similar trend is also observed in Figure 6b, which illustrates cortical features in the source space.

Further comparison between Figure 6a,b reveals that the mean effect size of local efficiency increases by 0.006, and the statistical significance of peak value differences is also enhanced. Specifically, local efficiency not only shows a significant increase in the gamma band (*p* < 0.0001) but also demonstrates significant differences across the remaining three bands (theta: *p* < 0.0001, alpha: *p* < 0.0001, beta: *p* < 0.0001). Similarly, the significance of peak value differences strengthens across all four bands (all *p*-values < 0.0001). These findings indicate that brain cortical features derived from the Desikan–Killiany atlas provide stronger discriminative power in group comparisons. This further suggests that source-space cortical features offer higher sensitivity and robustness in capturing depression-related neurophysiological differences.

**Figure 7:**
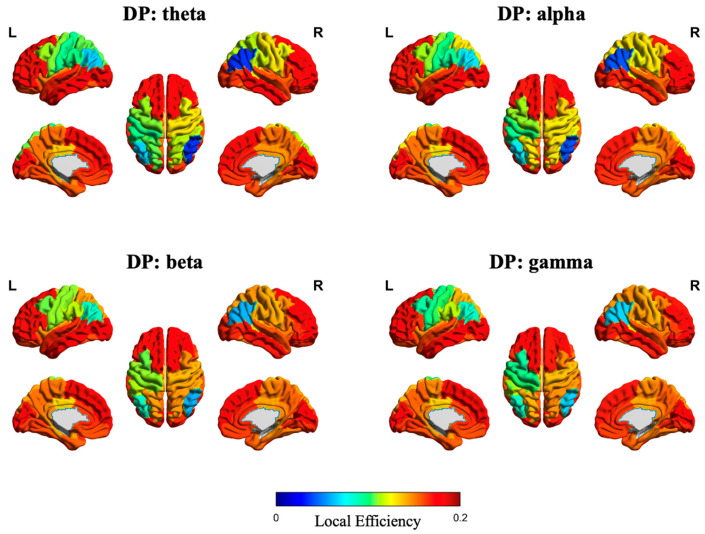
Source-space distribution of local efficiency in the DP group. The four subplots correspond to brain topographic maps for the theta, alpha, beta, and gamma frequency bands.

**Figure 8:**
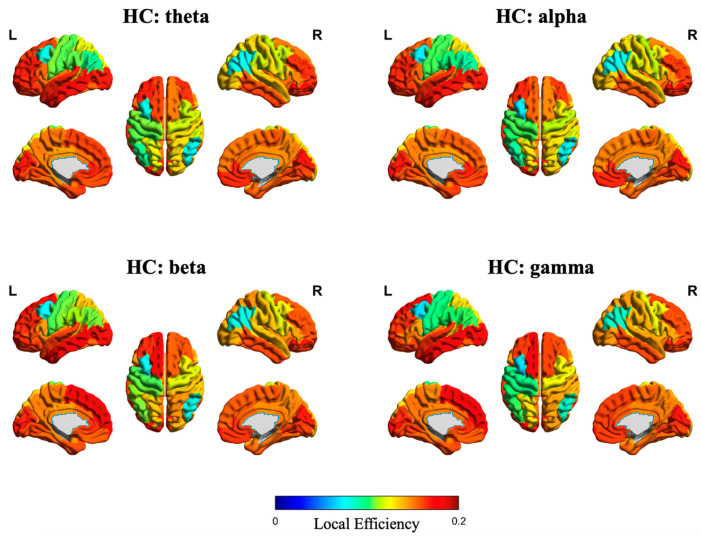
Source-space distribution of local efficiency in the HC group. The four subplots correspond to brain topographic maps for the theta, alpha, beta, and gamma frequency bands.

**Figure 9:**
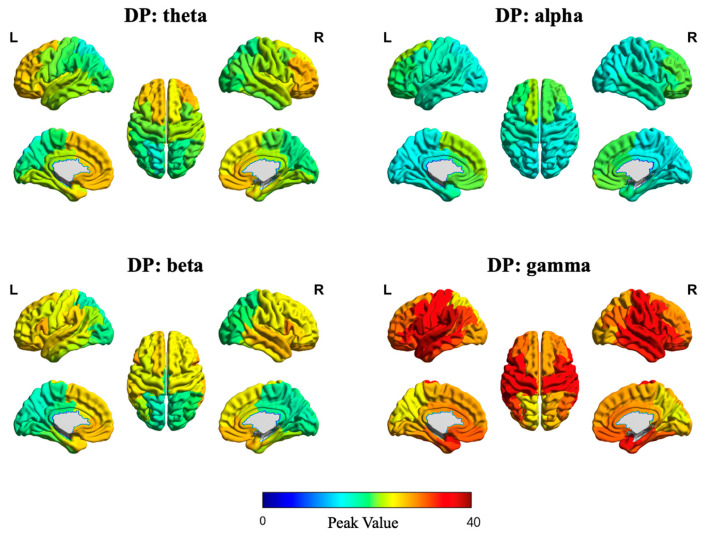
Source-space distribution of peak value in the DP group. The four subplots correspond to brain topographic maps for the theta, alpha, beta, and gamma frequency bands.

**Figure 10:**
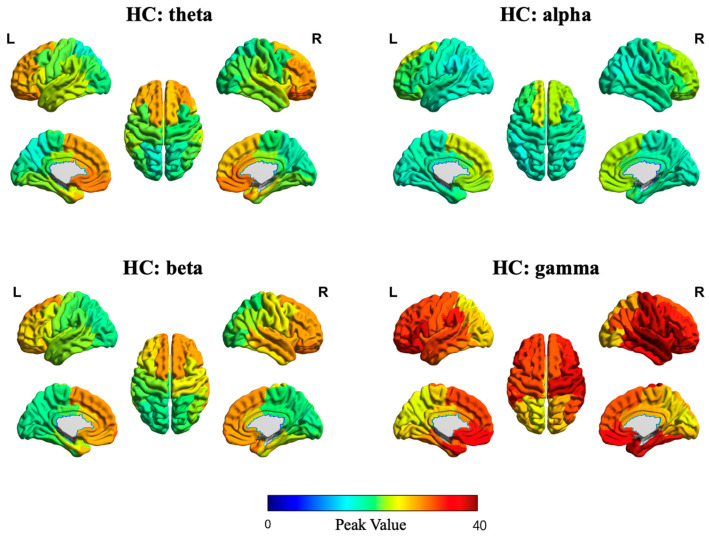
Source-space distribution of peak value in the HC group. The four subplots correspond to brain topographic maps for the theta, alpha, beta, and gamma frequency bands.

In addition, Figure 7 and Figure 8 illustrate the source-space local efficiency distributions of the DP and HC groups, respectively, across four frequency bands, while Figure 9 and Figure 10 depict the peak value distributions for the same groups and frequency bands. In all brain topographic maps, warm colors indicate higher feature values, and cool colors indicate lower feature values.

In summary, brain cortical features reconstructed in the source space outperform traditional scalp signals across multiple feature dimensions, further providing physiological support and explanation for the improved classification performance observed in Section 3.5.

## 4. Conclusions

In this paper, we propose a multi-stage deep learning model for the EEG-based depression classification, aiming to effectively mitigate the challenges of volume conduction effect and sample imbalance. By integrating the CFE, FA, GCN, and FADA modules, the model achieves superior classification performance compared to existing methods on the PRED+CT dataset, reaching an accuracy of 85.33%. Notably, the CFE strategy significantly enhances the discriminative ability of the features, while the consistent group-level differences observed across multiple frequency bands in source-space features further support the model’s neurophysiological plausibility. Overall, the proposed method provides an efficient and interpretable solution for developing robust EEG-based depression classification models.

## 5. Limitations

Although the proposed multi-stage deep learning model demonstrates promising classification performance, several limitations warrant further improvement. First, while sLORETA is employed for source-space reconstruction, it remains limited in terms of source localization accuracy and robustness to noise. Future research could explore Bayesian inference or data-driven source localization methods to enhance the physiological plausibility of cortical activity mapping. Second, the FADA module primarily addresses inter-class imbalance but does not explicitly model intra-class heterogeneity, which may affect the model’s ability to classify boundary or ambiguous samples. Finally, although the FA module effectively captures spatiotemporal dependencies in EEG signals, it underutilizes region-specific information related to brain function, structure, or signal characteristics. Incorporating regional priors or functional annotations in future work may further improve the model’s capacity to identify disease-relevant cortical patterns.

## Figures and Tables

**Table 1:** Left hemisphere regions based on the Desikan–Killiany atlas.

ID	ROI	ID	ROI
1	Banks of superior temporal sulcus	18	Pars orbitalis
2	Caudal anterior cingulate	19	Pars triangularis
3	Caudal middle frontal	20	Pericalcarine
4	Cuneus	21	Postcentral
5	Entorhinal	22	Posterior cingulate
6	Fusiform	23	Postcentral
7	Inferior parietal	24	Precuneus
8	Inferior temporal	25	Rostral anterior cingulate
9	Isthmus cingulate	26	Rostral middle frontal
10	Lateral occipital	27	Superior frontal
11	Lateral orbitofrontal	28	Superior parietal
12	Lingual	29	Superior temporal
13	Medial orbitofrontal	30	Supramarginal
14	Middle temporal	31	Frontal pole
15	Parahippocampal	32	Temporal pole
16	Paracentral	33	Transverse temporal
17	Pars opercularis	34	Insula

**Table 2:** Extracted features and their descriptions.

Dimension	Feature	Description
Spatial	Clustering Coefficient	The closeness between neighboring nodes.
	Local Efficiency	The communication efficiency between neighboring nodes
Temporal	Peak Value	Maximum value of the signal.
	Skewness	The symmetry of the signal distribution.
Spectral	Band Power	The power is calculated as the signal Power Spectral Density.
	Relative Power	Relative power is calculated as the absolute power in a given frequency band normalized to the total power.
Nonlinear	Sample entropy	The unpredictability of the signal.
	Fuzzy entropy	The unpredictability of the signal, emphasizing the continuity of local features.

**Table 3:** Demographic information and subscale scores from the BDI and the TAI.

Information	DP (*n* = 46)	HC (*n* = 74)	*p*-Value ^1^
Gender (male/female)	12/34	34/40	0.03
Age (mean ± std)	18.73 ± 1.14 ^2^	18.97 ± 1.21	0.30
BDI (mean ± std)	22.21 ± 4.89	1.74 ± 1.66	<0.0001
TAI (mean ± std)	55.76 ± 7.08	31.14 ± 5.46	<0.0001

^1^ *p*-value is derived from independent-samples *t*-tests between DP and HC. ^2^ std denotes standard deviation.

**Table 4:** Parameters settings.

Parameters	Our Model
Batch Size	16
Learning Rate	5 × 10^−5^
Early Stopping Patience	15
Dropout Rate	0.3
Optimizer	Adam
Epoch	70
Number of attention heads	4
Hidden dimension	512

**Table 5:** Comparison with other existing models on PRED+CT dataset.

Model	ACC (%)	F1-Score (%)	REC (%)	PRE (%)
Lawhern et al., 2018 [55]	65.62	77.03	**97.38** *	63.82
Tang et al., 2021 [56]	65.34	76.42	94.93	64.02
Ding et al., 2023 [57]	77.70	82.39	88.47	77.22
Luo et al., 2024 [58]	77.78	82.75	90.23	76.46
Zhang et al., 2024 [52]	83.17	81.74	84.15	82.93
Our	**85.33**	**83.42**	85.56	**84.05**

* Bold values indicate the highest performance for ACC, F1-score, REC, and PRE, respectively.

**Table 6:** Ablation experiments for CFE, FA, and FADA modules. The symbol “✓” indicates that the module is included, while “–” denotes its removal. For clarity and readability, models under different experimental configurations are labeled as S1–S8.

Index	CFE	FA	FADA	ACC (%)	F1-Score (%)	REC (%)	PRE (%)
S1.	–	–	–	75.81	72.61	67.48	79.32
S2.	✓	–	–	79.80	77.15	75.07	81.31
S3.	–	✓	–	77.50	74.56	70.73	80.17
S4.	–	–	✓	77.00	73.96	69.74	79.91
S5.	✓	✓	–	83.50	81.33	82.07	83.14
S6.	✓	–	✓	83.00	80.77	81.13	82.89
S7.	–	✓	✓	79.00	76.23	73.54	80.90
S8.	✓	✓	✓	85.33	83.42	85.56	84.05

## Data Availability

The dataset used in this study is publicly available at https://openneuro.org/datasets/ds003478/versions/1.1.0 (accessed on 7 March 2025). Furthermore, the data were preprocessed using the EEGLAB toolbox (https://sccn.ucsd.edu/eeglab/index.php (accessed on 5 March 2025)) in MATLAB 2019a (MathWorks, Inc., Natick, MA, USA). The processing code can be obtained from the corresponding author upon reasonable request.

## Abbreviations

The following abbreviations are used in this manuscript:

ACC	Accuracy
BDI	Beck Depression Inventory
CFE	Cortical Feature Extraction
Cp	Clustering Coefficient
DP	Depression Patients
EEG	Electroencephalography
FA	Feature Attention
FADA	Focal Adversarial Domain Adaptation
fMRI	functional Magnetic Resonance Imaging
FN	False Negatives
FP	False Positives
FPR	False Positive Rate
GCN	Graph Convolutional Network
HC	Healthy Controls
PET	Positron Emission Tomography
PLV	Phase Locking Value
PRE	Precision
REC	Recall
ROI	Region of Interest
sLORETA	standardized Low-Resolution Brain Electromagnetic Tomography
SVM	Support Vector Machine
TAI	Trait Anxiety Inventory
TN	True Negatives
TP	True Positives
TPR	True Positive Rate
